# Donor-Like Surface Traps on Two-Dimensional Electron Gas and Current Collapse of AlGaN/GaN HEMTs

**DOI:** 10.1155/2013/931980

**Published:** 2013-11-18

**Authors:** Chen-hui Yu, Qing-zhou Luo, Xiang-dong Luo, Pei-sheng Liu

**Affiliations:** ^1^Jiangsu Key Laboratory of ASIC Design, Nantong University, Nantong 226019, China; ^2^School of Remote Sensing, Nanjing University of Information Science and Technology, Nanjing 210044, China; ^3^Key Laboratory of Nanodevices and Applications, Suzhou Institute of Nano-Tech and Nano-Bionics, Chinese Academy of Sciences, Suzhou 215123, China

## Abstract

The effect of donor-like surface traps on two-dimensional electron gas (2DEG) and drain current collapse of AlGaN/GaN high electron mobility transistors (HEMTs) has been investigated in detail. The depletion of 2DEG by the donor-like surface states is shown. The drain current collapse is found to be more sensitive to the addition of positive surface charges. Surface trap states with higher energy levels result in weaker current collapse and faster collapse process. By adopting an optimized backside doping scheme, the electron density of 2DEG has been improved greatly and the current collapse has been greatly eliminated. These results give reference to the improvement in device performance of AlGaN/GaN HEMTs.

## 1. Introduction

Two-dimensional electron gas (2DEG) based AlGaN/GaN high electron mobility transistors (HEMTs) have shown promising advantages in high-power, high-frequency, and high-temperature applications [1–4]. They have also demonstrated good properties in optoelectronic responses [5]. The drain current collapse effect in these devices is a serious obstacle at the present stage to further improve device performances [6–8]. Some efforts have been made to explore the mechanisms of drain current collapse, such as self-heating [9–11], trapping [3], and surface states [12–15]. The bulk traps in AlGaN/GaN layers which absorb electrons from channels and virtual gate effects [12] which deplete the channel in the device by the accumulated negative charges in the surface have been found to be the main reason causing the reduction of 2DEG in channels [16]. However, the influence of donor-like surface traps on the drain current collapse has not been fully discussed. Donor-like traps mainly originate from shallow impurities, interface states, and surface states. They may interact with the negative charges in the surface which have a decisive influence on the 2DEG in the HEMT channels [12–16]. The effects of donor-like surface traps with different energy levels and density have been ignored in previous work.

In this paper, we analyze these ignored effects in AlGaN/GaN HEMTs with a two-dimensional device simulation method. We adopt the density of the surface charge as a measurement of the activities of surface traps, since filling or emptying charges on the surface from the traps can change the charge density on the surface directly and influence the response of the channel electrons to the voltage consequently. The sensitivity of current collapse to the variation of negative charges on the surface is firstly investigated for a better understanding of behavior of donor-like surface traps.

## 2. Method and Device Structure

**Figure 1:**
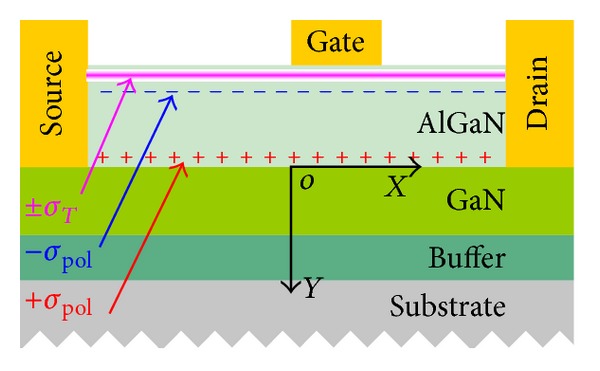
Cross-sectional structure of AlGaN/GaN HEMT. Positive sheet charge +*σ*_pol_ is caused by spontaneous polarization and piezoelectric effect. Equivalent negative sheet charge −*σ*_pol_ is fixed on the AlGaN surface. Surface trap states are represented with ±*σ*_*T*_.

Two-dimensional drift-diffusion simulations of the AlGaN/GaN HEMTs are performed. Device structure of the AlGaN/GaN HEMT is shown in Figure 1. The gate length is 0.7 *μ*m and the opening spaces between the contacts are *L*_GD_ = 0.7 *μ*m and *L*_SG_ = 2 *μ*m. The thickness of AlGaN layer with composition of 35% aluminum is 29 nm. The mobilities of electrons and holes in GaN layer are 100 cm^2^ V^−1^ s^−1^and 30 cm^2^ V^−1^ s^−1^, respectively. The mobilities of electrons and holes in AlGaN layer are 100 cm^2^ V^−1^ s^−1^ and 5 cm^2^ V^−1^ s^−1^, respectively [9, 17–21].

A positive sheet charge +*σ*_pol_ caused by spontaneous polarization and piezoelectric effect [22–24] is fixed at the interface and the equivalent negative sheet charge −*σ*_pol_ on the AlGaN surface. The fixed sheet charge density is assumed to be −1.15 × 10^13^ cm^−2^ on the AlGaN surface and 1.15 × 10^13^ cm^−2^ at AlGaN/GaN interface [25–27], respectively. Surface states *σ*_*T*_ are considered uniformly distributed on the regions between source and gate. When the surface states are taken into calculation, the initial charge density is modified by adding or removing static charges at the surface. The net charge density between the contacts is expressed as *σ*_net_ = −*σ*_pol_ + *σ*_*T*_. A default temperature of 300 K is employed in simulations [28]. A transient voltage *V*_dd_ = 6 V is applied to the drain with maintaining gate bias *V*_*G*_ = 0 V. The drain voltage is pulsed from 0.1 V to *V*_dd_ and the pulse time is adjusted in each case. 

## 3. Results and Discussion

**Figure 2:**
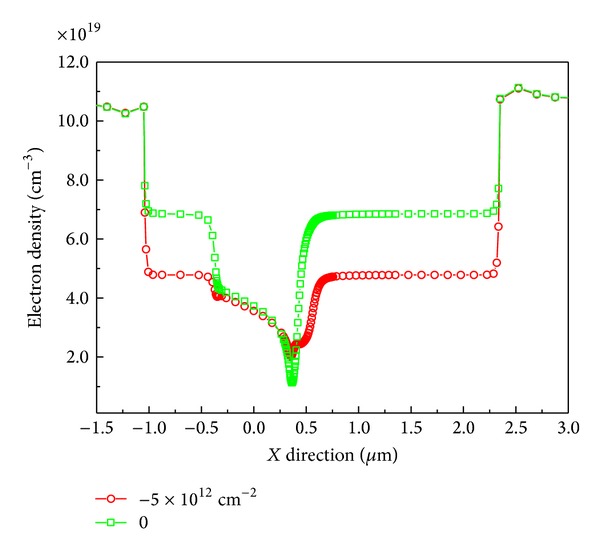
Electron density versus *X* direction cut at AlGaN/GaN interface. A depletion of 2DEG in channel due to negative charges on the surface is shown.

In Figure 2, the reduction of average electron density in the channel is shown as the surface charges, *σ*_*T*_, change from 0 to −5 × 10^12^ cm^−2^. The increase of negative charge density (NCD) on the surface leads to the depletion of 2DEG. This is mainly caused by the induced positive charges that appeared around the AlGaN/GaN interface by NCD, which then neutralize the electrons in channel. Another point is the same increase of electron density near the gate along *X* direction as the previous increase of NCD. It provides clear evidence of the existence of a virtual gate in this device.

**Figure 3:**
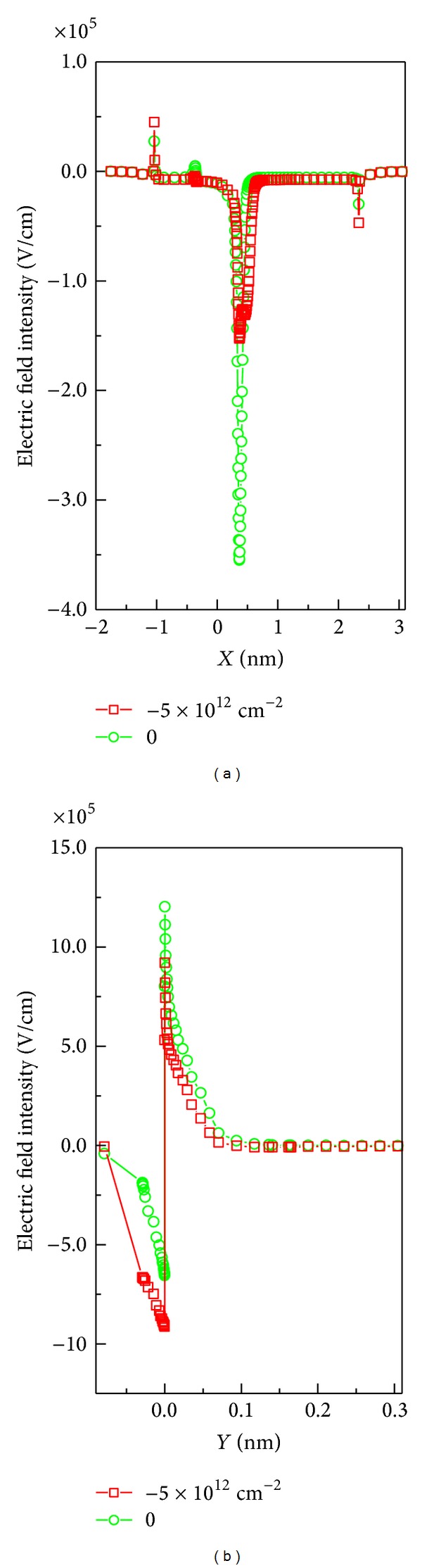
(a) Electric field intensity versus *X* direction. (b) Electric field intensity versus *Y* direction.

The electric field intensities in both *X* and *Y* direction as the surface charges, *σ*_*T*_, change from 0 to −5 × 10^12^ cm^−2^ are shown in Figure 3. The electric field intensity in *X* direction is cut at AlGaN/GaN interface, *Y* = 0 nm, and in *Y* direction is cut at *X* = 0.25 nm, as shown in Figure 1. The electric field intensity in both *X* and *Y* directions decreases as the negative surface charge density is increased. The decrease of electric field intensity is consistent with the decrease of electron density in Figure 2 and produces positive consequences. Firstly, since the electric field intensity near the interface is reduced, the electrons in the channel cannot acquire high enough temperature, and the number of hot electrons reduces. Moreover, the addition of negative charges at surface makes surface potential lower and interface potential higher [28, 29], which in turn causes the electron affinity to increase. The higher electron affinity causes again the reduction of the amount of hot electrons. Secondly, the quantum tunneling effect is weakened with the increase of potential barrier and the decrease of electron energy [30]. The weakening of both hot electron and quantum tunneling effects prevents electrons escaping from the channel. Therefore, the increase of the negative charges on the surface is helpful in eliminating the drain current collapse.

**Figure 4:**
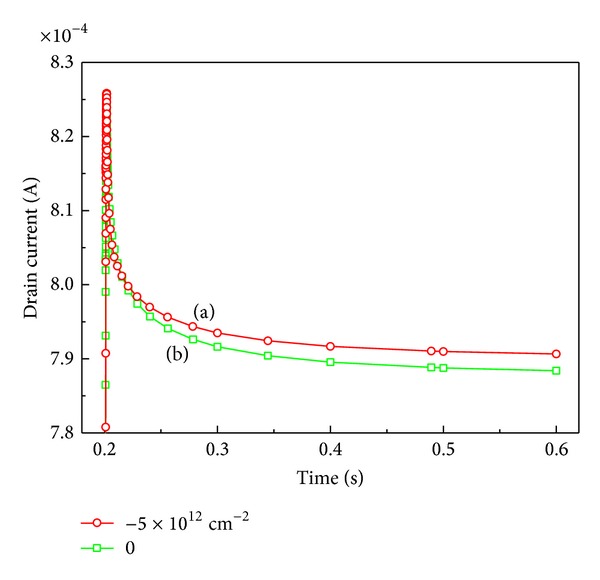
Comparison of drain current versus pulse time. The curves of drain current are shifted to hold two peaks together for better comparison.

The comparison of drain current collapse under different surface negative charges is shown in Figure 4 with a pulse time of 2 ms. One open circle is with 5 × 10^−12^ cm^−2^ negative charges added at surface and another open square is with no negative charges. The peaks of the time-dependent drain current under different amount of surface negative charges are put together in order to have a better understanding of the decay process of drain current. We can see that the collapse of drain current in line (a) (with −5 × 10^12^ cm^−2^ in the surface) is reduced because of the addition of negative charges at AlGaN layer surface. The negative charges make the electrons more difficultly escape from the channel. Besides, the drain current in the line (a) takes less time to reach the steady state. In other words, the process of drain current collapse with more negative charges is accelerated. 

**Figure 5:**
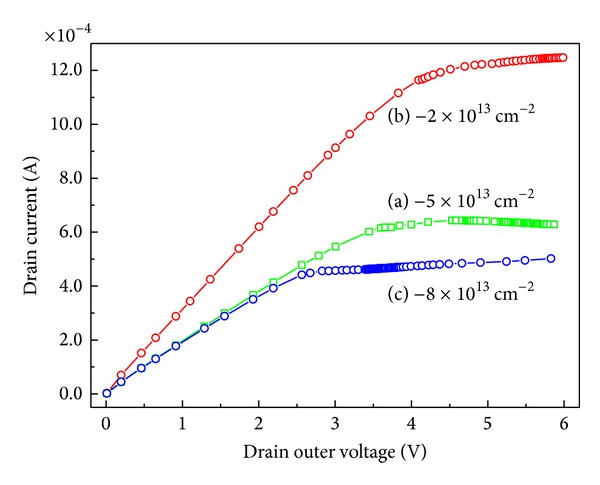
Drain current response versus drain voltage under different amount of surface charges.

The drain current response versus drain voltage under different amount of surface charges is shown in Figure 5. Taking case (a) as a standard example, cases (b) and (c) can be considered as added 3*σ*_pol_ positive charges and 3*σ*_pol_ negative charges on the surface of case (a), respectively. When the negative charge −3*σ*_pol_ is added to the surface as case (c), the drain current does not decrease as much as the increase of the drain current caused by the same amount of +3*σ*_pol_ positive charge added at the surface as case (b). This result means that the transient drain current response to time of AlGaN/GaN HEMTs is more sensitive to the presence of the positive charges rather than the negative charges. In the HEMT device, the holes are the main origin of great difference in sensitivity to the charge changes at the surface. The free holes populate at the surface, and part of them is caused by the inner electric field because the inner electric field divides a couple of electrons and holes into two individual parts. Then, these holes come to the surface, and part of the holes gathers at the surface because they get to the surface to compensate the extra negative charge added in the surface and maintain the electric neutrality. Therefore, when the negative charges are added at the surface, they do not reduce the drain current because some of them are neutralized by holes in the surfaces first.

**Figure 6:**
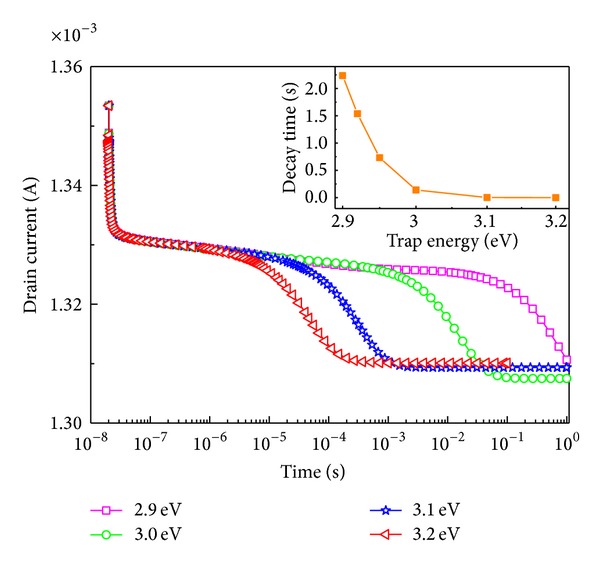
Drain current collapses at different energy levels. Inset is the curves of decay time to steady states.

Transient drain current responses versus time with *V*_gs_ = 0 V and a fixed trap density of 4 × 10^13^ cm^−2^ are shown in Figure 6. Transient voltages are applied to drains. The time for the voltage to turn on is about 10^−8^ s. The energy levels of the donor type traps are referenced to the edge of valence band and set to be 3.2 eV, 3.1 eV, 3.0 ev and 2.9 eV, respectively. For better comparison, the different peak values of the drain currents are hold together. A noticeable decrease in the decay value of drain current can be observed as the donor-like trap energy level goes higher. The increase of DTI (donor-like trap ionization) leads to the increase of number of positive charges accumulating in the surface. When the drain voltage jumps up from 0 V to 6 V, millions of electrons are injected into the drain electrode and then couple with these positive charges. This process causes the decrease of positive charge density (PCD). We can consider the decrease of PCD as addition of negative charges in the surface. From discussion above, we know that the addition of negative charges weakens not only the hot electron effects, but also the quantum tunneling effects. Therefore, the collapse amount is reduced as the energy level goes higher. 

Meanwhile, the noticeable decrease in the decay time is also observed as the donor-like trap energy level goes higher. Higher energy level traps produce more positive charges and then these charges are neutralized by injected electrons. As more negative charges are added on the surface, depletion process is accelerated, and thus the transient drain current costs less time to reach the steady states. The decay time with different donor-like trap energies is shown in the inset. The decay time does not change linearly with the change in the energy level but decreases exponentially along with the increase in donor-like traps energy level. As the energy level becomes higher, the differences of decay times become smaller. This is also the result of change of surface charges. The higher energy levels cause more positive charges to populate at the surface and then these positive charges are partially captured by electrons. It means that more negative charges are added at the surface. Similar to the discussion above, the drain current response is not sensitive to the presence of the negative charges. Thus, after the appearance of more negative charges at the surface, the current collapse process is weakened.

**Figure 7:**
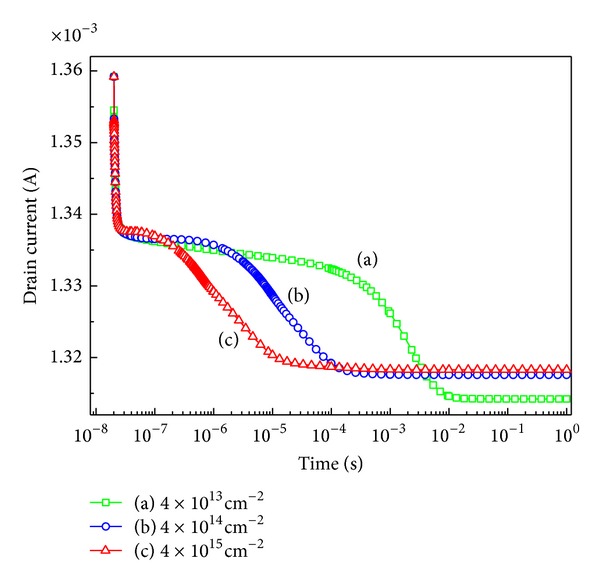
Drain current collapse at different trap density.

The transient response at different values of trap density on the surface with a fixed trap energy level 3.1 eV is shown in Figure 7. The trap densities are considered with (a) *σ*_*T*_ = 4 × 10^13^ cm^−2^, (b) *σ*_*T*_ = 4 × 10^14^ cm^−3^, and (c) *σ*_*T*_ = 4 × 10^15^ cm^−2^. The differences of decay time become smaller with increasing trap density. Similar to the explanation of Figure 6, it can be concluded that the variation of charge in the surface has great role in the current response. The trap density of (c), namely, 4 × 10^15^ cm^−2^, has the most positive charge density at the surface. After the voltage is shifted from 0.1 V to 6 V, the electrons injected from drain electrode are captured by the positive charges. The process causes negative charge density to be increased at the surface. Because the current response is less sensitive to the addition of negative charge density, the case (c) in which more negative charges are captured has less decay time compared to case (a). 

**Figure 8:**
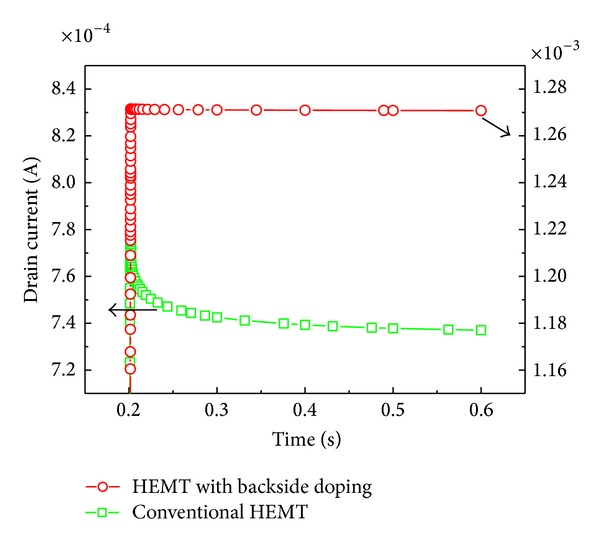
Comparison before and after backside doping.

To eliminate the negative influence of surface trap density on the surface, we suggest an optimizing scheme with backside doping [9, 29] to improve the performance of these HMETs. The total thickness of GaN layer is 75 nm, the bottom 35 nm is doped with phosphorus (2 × 10^18^ cm^−3^). The GaN layer of 75 nm can restrict electron effectively. The doping of the bottom modulates the energy band structure [9] and thus not only prevents the electron going into the bulk traps, but also makes a bigger potential well for holding more electrons. When considering the advantages of backside doping and the disadvantages of parasitic conductance, the doping concentration and the thickness of doping layer have to be optimized as previously reported work [9, 29].  Figure 8 shows the comparison of drain current collapse before and after backside doping. It can be seen that the drain current has been enhanced and the collapse in drain current has almost been eliminated. After introducing the backside doping, we can improve the electron density in the device and eliminate the drain current collapse effect greatly.

## 4. Conclusions 

The effects of donor-like surface traps on current collapse of AlGaN/GaN HEMTs have been systematically investigated. It is demonstrated that the negative charges have been neutralized by the positive charges on the surface. Thus, the current response is less sensitive to the presence of the negative charge. Based on the response of drain current, the ionization of donor type traps is thought to cause the reduction of decay time and the acceleration of decrease of drain current. The ionization process collects holes and then induces an increase in the number of negative charges gathering on the surface. Higher energy levels or larger density of donor type traps leads to the increase of ionization of donor type traps in the surface and subsequently causes the reduction of decay time and the weakening of current collapse amount. By adopting the backside doping in the bottom of GaN layer with 50 nm thickness, the energy band structure can be regulated and more electrons can be accumulated in the channel. Then, the collapse of drain current has been eliminated effectively. Our results are beneficial to the improvement of drain current in AlGaN/GaN HEMTs.
